# Potential Peripartum Markers of Infectious-Inflammatory Complications in Spontaneous Preterm Birth

**DOI:** 10.1155/2015/343501

**Published:** 2015-05-18

**Authors:** Vojtech Tambor, Marie Vajrychova, Marian Kacerovsky, Marek Link, Petra Domasinska, Ramkumar Menon, Juraj Lenco

**Affiliations:** ^1^Biomedical Research Center, University Hospital Hradec Kralove, Sokolska 581, 50005 Hradec Kralove, Czech Republic; ^2^Institute of Molecular Pathology, Faculty of Military Health Sciences, University of Defence, Trebesska 1575, 50001 Hradec Kralove, Czech Republic; ^3^Department of Obstetrics and Gynecology, Faculty of Medicine in Hradec Kralove, Charles University in Prague, Simkova 870, 50005 Hradec Kralove, Czech Republic; ^4^Division of Maternal-Fetal Medicine & Perinatal Research, Department of Obstetrics and Gynecology, The University of Texas Medical Branch at Galveston, 301 University Boulevard, MRB 11-138, Galveston, TX 77555, USA

## Abstract

Spontaneous preterm birth significantly contributes to the overall neonatal morbidity associated with preterm deliveries. Nearly 50% of cases are associated with microbial invasion of the amniotic cavity followed by an inflammatory response. Robust diagnostic tools for neonates jeopardized by infection and inflammation may thus decrease the overall neonatal morbidity substantially. Amniotic fluid retrieved during labor retains fetal and pregnancy-related protein fingerprint and its sampling does not place any unwanted stress on women. Using exploratory and targeted methods we analyzed proteomes of amniotic fluid sampled at the end of spontaneous preterm labor prior to delivery from women with and without infection and inflammation. Exploratory data indicated several amniotic fluid proteins to be associated with infectious-inflammatory complications in spontaneous preterm birth. LC-SRM analysis subsequently verified statistically significant changes in lipocalin-1 (*P* = 0.047 and AUC = 0.67, *P* = 0.046), glycodelin (*P* = 0.013 and AUC = 0.73, *P* = 0.013), and nicotinamide phosphoribosyltransferase (*P* = 0.018 and AUC = 0.71, *P* = 0.01).

## 1. Introduction

Despite the progress in perinatal medicine and the increasing knowledge on the risk factors and underlying mechanisms associated with preterm birth (i.e., childbirth occurring in less than 37 completed weeks of gestation [1]), its rates still range between 5 and 9% in Europe and other developed countries [2], and, in the USA, the rate had risen to 12% in 2009 [3, 4]. Preterm birth is the leading cause of perinatal mortality and is associated with rates of long-term morbidity such as cerebral palsy, developmental delay, retinopathy of prematurity, and others in up to 75% of preterm children [2, 5]. Preterm birth can occur either iatrogenically or spontaneously, which further encompasses (i) preterm prelabor rupture of membranes and (ii) spontaneous preterm labor and delivery with intact membranes [6], hereafter referred to as spontaneous preterm birth (sPTB) [6]. sPTB, which is the subject of the present work, accounts for at least 50% of all preterm births [7–9].

Several studies have shown that microbial invasion of the amniotic cavity (MIAC) is one of the risk factors for spontaneous preterm labor [10]. MIAC followed by intra-amniotic inflammation may result in histological chorioamnionitis (HCA), which manifests as infiltration of the placenta, fetal membranes, and umbilical cord by neutrophils and other immunocompetent cells. There is a solid body of evidence demonstrating that the presence of both these conditions is associated with significant neonatal morbidity, having short- and long-term consequences on the quality of life [11]. Therefore, there is an urgent need for robust diagnostic tests to identify women and newborns who may benefit from specific perinatal and postnatal interventions.

During the last decade, mass spectrometry-based proteomics has become a valued tool for conducting comprehensive analyses in biological and clinical samples [12]. However, even with the latest instrumentation, it is still challenging to describe changes in very low-abundance proteins, particularly in body fluids, which represent the most promising diagnostic material. To avoid the discovery of repeatedly observed high-abundance proteins as putative markers, prefractionation and separation steps must precede mass spectrometry analyses [13, 14]. As these analyses are time consuming, biomarker discovery studies are often performed employing a limited number of representative clinical samples or using a pooling strategy. However, without subsequent verification of primary discovery findings through accurate quantitative assays, the results obtained from such analyses are supported only by restricted significance.

The method of choice for conducting accurate and sensitive measurements of protein concentrations in clinical samples is to use antibody-based assays, such as ELISAs. Unfortunately, antibodies or validated immunoassays for new marker candidates are often not readily available. SRM is a versatile LC-MS/MS based technique with the potential to achieve accurate and fairly sensitive protein quantification [15]. SRM assays are intrinsically easy to multiplex, such that tens of different proteins may be monitored in a single analysis, which reduces sample consumption. As an SRM assay can be developed relatively quickly, this technique is being eagerly adopted for rapid initial verification of primary exploratory proteomic findings. Recently, dimethyl labeling was shown to be able to substitute synthetic stable isotope labeled peptides as internal standards for LC-SRM assays, representing fast and cost-effective alternative for accurate relative quantification of large number of analytes [16].

Here, we perform exploratory and targeted proteomic analyses of amniotic fluid to reveal and verify proteins potentially useful for detection of newborns jeopardized by intra-amniotic infection and inflammation. The samples were collected at the end of the first stage of sPTB followed by delivery in women with and without both MIAC and HCA. Amniotic fluid retrieved during labor represents a reasonable diagnostic material as it fully retains a fetal and pregnancy-related protein fingerprint, but in contrast to transabdominal amniocentesis, the method and timing of the collection do not place any unwanted stress on the mother. To the best of our knowledge our study represents the first attempt of a comprehensive proteomic analysis of this unique clinical specimen.

## 2. Materials and Methods

### 2.1. Amniotic Fluid Samples

Samples were collected between 2008 and 2011 as a part of a major study to examine the genetic and biomarker changes associated with sPTB and to determine the factors involved in the racial disparity between African Americans and Caucasians. This study was approved by the Institutional Review Board of Tristar Nashville and the Western Institutional Review Board of the Perinatal Research Center, Nashville, TN. All samples were collected after obtaining written informed consent from all participants.

All of the women included in the study were recruited at the Department of Obstetrics and Gynecology of Centennial Women's Hospital, Nashville, TN, USA. Amniotic fluid samples were collected from the forebag via needle puncture of intact membranes at the end of first stage of labor. Amniotic fluid was centrifuged immediately for 10 min at 2,000 g and aliquots were stored at −70°C until analysis. Samples were supplemented with protease inhibitors (Roche Diagnostics, Basel, Switzerland) and filtered through a 0.22 *μ*m filter (TPP, Trasadingen, Switzerland).

sPTB was defined as the occurrence of regular uterine contractions at a minimum frequency of two contractions* per* 10 minutes, along with cervical changes, leading to delivery before the 37th week of gestation was completed. Women with the following criteria were enrolled in this cohort: a singleton pregnancy, Caucasian race, maternal age between 18 and 35 years, and no ultrasound signs of intrauterine growth restriction. The exclusion criteria were preterm prelabor rupture of membranes, preeclampsia, placenta previa, fetal anomalies, and/or medical/surgical complications of pregnancy.

MIAC was determined based on a positive aerobic/anaerobic cultivation of amniotic fluid or PCR detection of bacterial 16s rRNA. Histological examination of the placenta, the fetal membranes, and the umbilical cord was performed in all cases. The degree of polymorphonuclear leukocyte infiltration was assessed separately in the free membranes, in the placenta, and in the umbilical cord. The diagnosis of HCA was conducted based on the presence of diffuse neutrophil infiltration in the chorion decidua, chorionic plate, amnion, and umbilical cord.

### 2.2. Chemicals

All chemicals were from Sigma Aldrich in the highest grade available if not indicated otherwise (Sigma, St. Louis, MO). The solvents used for HPLC with UV detection were of HPLC grade, and those used for HPLC hyphenated to a mass spectrometer were of LC-MS grade (Sigma).

### 2.3. Sample Preparation and Immunoaffinity Removal of Ballast Proteins for Exploratory iTRAQ Analysis

**Figure 1:**
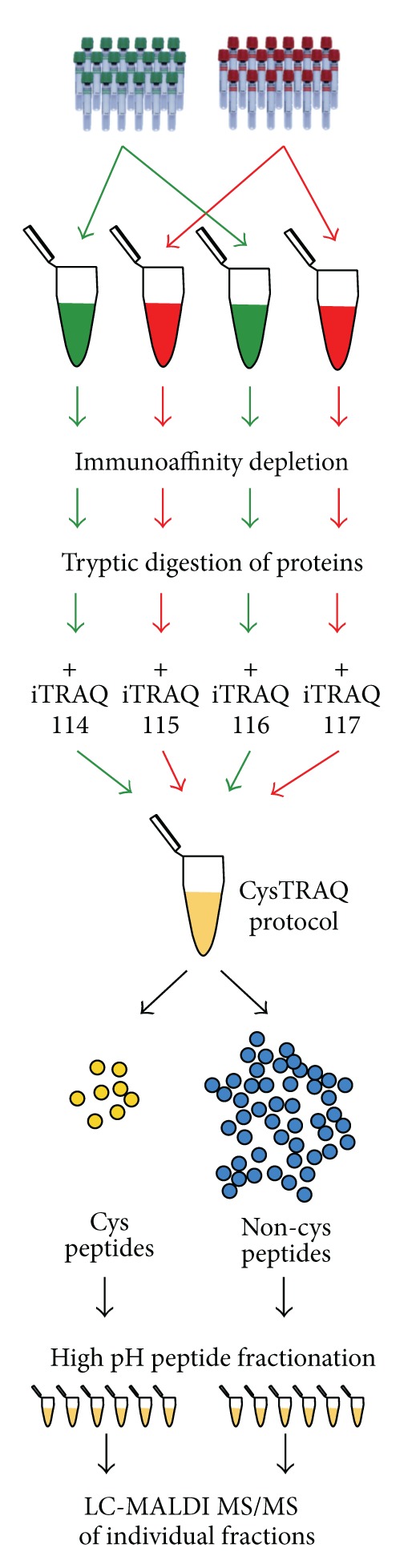
Sample processing for the exploratory iTRAQ analysis. Representative MIAC and HCA-positive and negative samples were created and processed as two duplicates. Samples were depleted from 14 high-abundance proteins and digested. Resulting peptides were iTRAQ labeled, combined, and processed according to the CysTRAQ protocol. Cysteinyl and noncysteinyl iTRAQ peptides were further fractionated using high pH reversed-phase chromatography. Eventually, each fraction was analyzed using LC-MALDI-MS/MS.

An overview on how the samples were processed for exploratory iTRAQ analysis is depicted in Figure 1. Protein concentrations were determined using a bicinchoninic acid assay (BCA) in each of the 31 MIAC and HCA-positive and 26 negative samples. An equal amount of protein was taken from each sample to create a representative MIAC and HCA-positive sample and a representative negative sample; both were created in duplicate. Each representative sample contained 2 mg of protein. The volume was adjusted to 4 mL using Multiple Affinity Removal System (MARS) buffer A (Agilent, Palo Alto, CA), and the samples were concentrated using Amicon Ultra filters (Millipore, Bedford, MA) with a 3 kDa cut-off. The retentates were adjusted to 200 *μ*L with MARS buffer A.

The 14 most abundant plasma proteins that could be anticipated to also be present in amniotic fluid at high concentrations were removed from the prepared representative samples using a MARS Hu-14 column (Agilent) in an Alliance 2695 HPLC system (Waters, Milford, MA), according to the manufacturer's instructions. MARS buffer A was exchanged three times for water using 3 kDa cut-off Amicon Ultra filters. The retentates were collected, and the protein concentration was determined via BCA.

### 2.4. Trypsin Digestion and Application of CysTRAQ Protocol

From each depleted sample, 200 *μ*g of protein was brought to a volume of 40 *μ*L with 250 mM triethylammonium bicarbonate buffer, pH 8.5 (TEAB), containing 0.1% RapiGest (Waters). The proteins were reduced using 5 mM tris(2-carboxyethyl) phosphine hydrochloride (TCEP) for 1 h at 60°C and digested overnight at 37°C with trypsin (Promega, Madison, WI) at a 1 : 50 ratio. The CysTRAQ method for fractionation of iTRAQ-labeled peptides based on the presence of cysteine residues was applied exactly according to recently published workflow [13]. The MIAC and HCA-negative samples were labeled with 114 and 116 tags, while MIAC and HCA-positive samples were labeled with 115 and 117 tags.

### 2.5. iTRAQ Peptide Fractionation Using High pH Reversed-Phase Chromatography

Desalted cysteinyl and noncysteinyl peptide fractions were redissolved in 200 *μ*L of 20 mM ammonium formate (NH_4_FA), pH 10. Fractionation was performed using the Alliance 2695 HPLC system (Waters). Samples were injected onto a Gemini C18 2 × 150 mm column (Phenomenex, Torrance, CA) filled with 3 *μ*m, 110 Å particles. The peptides were separated using a linear gradient ranging from 5% ACN, 20 mM NH_4_FA, pH 10, to 55% ACN, 20 mM NH_4_FA, pH 10, over 62 min. The eluted peptides were collected between the 20th and 60th min, resulting in 16 collected fractions* per* sample. Each fraction was acidified with formic acid (FA) and vacuum dried.

### 2.6. LC-MS/MS Analysis of iTRAQ Peptides on MALDI-TOF/TOF

Each high pH reversed-phase fraction was dissolved in 40 *μ*L of 5% ACN, 0.1% TFA, following nano-LC peptide separation using the UltiMate3000 HPLC system (Dionex, Sunnyvale, CA). The peptides were trapped on a 0.3 × 5 mm *μ* precolumn filled with C18 PepMap, 5 *μ*m, 100 Å particles (Dionex) and subsequently separated in a 0.1 × 150 mm analytical NanoEase column filled with Atlantis C18, 3 *μ*m, 100 Å particles (Waters) using a linear gradient ranging from 5% ACN, 0.1% TFA to 50% of 80% ACN, 0.1% TFA over 85 min at a flow rate of 360 nL/min. The eluate was mixed at a 1 : 4 ratio postcolumn in a Probot fraction collector (Dionex) with 3 mg/mL CHCA (LaserBio Labs, Sophia-Antipolis, France) in 70% ACN, 0.1% TFA. Fractions were collected every 8 s for 60 min on an OptiTOF LC-MALDI plate. MALDI analysis was performed in a 4800 MALDI-TOF/TOF (AB Sciex). MS spectra were acquired across the *m*/*z* range of 800–4 000 using 625 laser shots* per* spectrum. A maximum of 12 precursors was chosen for fragmentation in each MS spectrum, starting with the weakest precursor. CID MS/MS spectra were acquired with using 3000 laser shots.

### 2.7. Analysis of LC-MS/MS Data of iTRAQ Peptides

The spectra were evaluated with ProteinPilot 2.0.1 software (AB Sciex) using the Paragon search algorithm, Pro Group algorithm, and the integrated FDR analysis function [17, 18]. The data were searched against the UniProtKB/Swiss-Prot database (downloaded in August 2012). The samples were described using the following parameters: sample type: iTRAQ 4plex (peptide labeled); Cys alkylation: methyl methanethiosulfonate (MMTS); digestion: trypsin; species:* Homo sapiens*. The processing was specified as follows: quantitate: on; bias correction: on; ID focus: biological modifications; search effort: thorough; detected protein threshold: 0.05 (10.0%). For FDR determination, the software automatically searched the data against a concatenated database that contained both the forward and reversed protein sequences. The intensities of the iTRAQ reporter ions were corrected using isotope correction factors supplied with the kit.

### 2.8. Sample Preparation and Immunoaffinity Removal of Ballast Proteins for LC-SRM Verification

**Figure 2:**
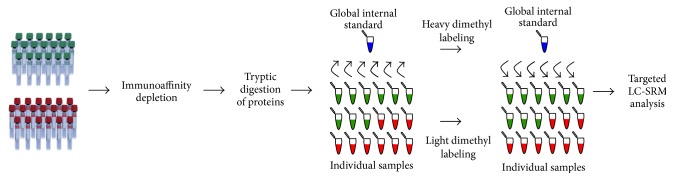
Sample processing for the LC-SRM verification. Each sample was subjected to immunoaffinity depletion of 14 high-abundance proteins and digested by trypsin. Portion of each sample was used to create a global internal standard (GIS). GIS peptides were dimethylated by formaldehyde-d_2_ whereas peptides in each sample were labeled by formaldehyde. GIS was spiked in each individual sample prior to LC-SRM analysis.

An overview on how the samples were processed for LC-SRM verification is depicted in Figure 2. A volume corresponding to 1 mg of protein was taken from each sample, except for five HCA and MIAC-positive samples and three HCA and MIAC-negative samples for which there was not enough material left. Immunoaffinity depletion in individual samples was performed as described above. MARS buffer A was exchanged three times for water using 3 kDa cut-off Amicon Ultra filters, and the final volume was adjusted with water to 140 *μ*L. The total protein concentration was determined via BCA.

### 2.9. Tandem Lys-C/Trypsin Digestion and Reductive Dimethyl Labeling for LC-SRM Verification

For each sample, 65 *μ*L of depleted material was mixed with 10 *μ*L of 1% RapiGest in 500 mM TEAB. Disulfides were reduced in 5 mM TCEP for 60 min at 60°C. Thiol residues were blocked in 10 mM MMTS for 10 min at room temperature. rLys-C (Promega) was added at 50 : 1 ratio. After six hours of incubation at 37°C, trypsin was added at 50 : 1 ratio, the volume was adjusted by water to 100 *μ*L, and samples were incubated overnight at 37°C. Samples were eventually split in two equal 50 *μ*L aliquots. One aliquot was subsequently used to generate a global internal standard (GIS).

Peptides in individual 50 *μ*L aliquots were dimethylated by incubation with 10 *μ*L of 4% formaldehyde and 10 *μ*L of 1 M sodium cyanoborohydride at 20°C for 15 min. Subsequently, pH was adjusted by adding 100 *μ*L of 100 mM sodium acetate, pH 5.2, exactly the same portion of the labeling reagents was added again, and samples were incubated at 20°C for 15 min. The GIS was labeled using 4% formaldehyde-d_2_ in equal reagents ratios as specified above. A small portion of GIS was saved for the development and refinement of the LC-SRM assay.

Individual light-labeled samples were mixed with an equal amount of GIS, acidified by TFA and incubated for 30 min at 37°C to hydrolyze RapiGest, which was subsequently removed via centrifugation. Samples were desalted using 3 M Empore C18-SD 4 mm/1 mL cartridges and vacuum dried. Prior to the analysis, samples were redissolved in 38 *μ*L of 5% ACN, 0.1% FA to get expected average concentration 0.5 *μ*g/*μ*L.

### 2.10. LC-SRM Assay Development

The LC-SRM assay development, including method refinement, and final data analysis were all performed in Skyline software (University of Washington, Seattle, WA) [19]. Randomly selected six MIAC and HCA-positive and six negative samples were mixed to create two development samples. These samples were digested and dimethylated using formaldehyde and sodium cyanoborohydride as described above.

First of all, development samples were used to build an MS/MS library of dimethylated peptides to facilitate and accelerate SRM transitions design. Each development sample (~1 *μ*g of peptides) was subjected to a nano-LC-MS/MS analysis. An UltiMate 3000 RSLCnano system controlled by Chromeleon software (both from Thermo Scientific) was used for chromatography separation. The trap column configuration consisted of PepMap100 C18, 3 *μ*m, 100 Å, 0.075 × 20 mm trap column and PepMap RSLC C18, 2 *μ*m, 100 Å, 0.075 × 150 mm analytical column (both from Thermo Scientific). The samples were loaded onto the trap column at 5 *μ*L/min of loading phase A (2% ACN, 98% water, and 0.05% TFA) for 5 min. Peptides were separated by a gradient formed by mobile phase A (water and 0.1% FA) and mobile phase B (80% ACN, 20% water, and 0.1% FA), running from 4 to 34% in 68 min and from 34 to 55% of mobile phase B in 21 min at a flow rate of 0.3 *μ*L/min at 40°C. Separation was monitored at 215 nm.

Eluted peptides were electrosprayed into a Q-Exactive mass spectrometer using a Nanospray Flex ion source (Thermo Scientific, Bremen, Germany). The full MS/Top12 experimental setup was used. Positive ion full scan MS spectra (*m*/*z* 300–1800) were acquired using a 1 × 10^6^ target in the Orbitrap at 70 000 resolution (at *m*/*z* 200). The lock mass of *m*/*z* 445.12003 ([(C_2_H_6_SiO)_6_+H]^+^) was used for internal calibration of mass spectra.

Precursor ions of charge state ≥ 2 and threshold intensity of 1 × 10^5^ counts were selected for HCD fragmentation, with an exclusion window of 60 s. The isolation window of 2 Da and normalized collision energy of 27 were used. Each product ion spectrum was acquired in the Orbitrap at 17 500 resolution, with a 1 × 10^6^ AGC target and a maximum 60 ms injection time.

MS/MS spectra were searched in Proteome Discoverer software (Thermo Scientific) using MASCOT engine (Matrix Science, London, UK). The tryptic specificity was set to full and two missed cleavages were allowed. The mass tolerance was set to 10 ppm for precursors and 20 mmu for fragment ions. Oxidized Met and dimethylation of N-terminus and of Lys were set as dynamic modifications while Cys thiomethylation was set as a fixed modification. MS/MS spectra explained with a cut-off score 0.80 were extracted by the Skyline software from the MASCOT results and stored in an MS/MS library.

The FASTA sequences of proteins of interest were retrieved from UniProt and imported into the Skyline. The following constraining criteria were applied on peptide selection: digestion: trypsin (cleavage at Arg or Lys unless Pro is the next amino acid); no missed cleavage allowed; peptide length: between 6 and 25 amino acids; excluding the first five N-terminal amino acids; structural modifications: methylthio at Cys and dimethylation of N-terminus and Lys. Peptides sharing sequence with additional human protein(s) were excluded; Leu and Ile were treated as interchangeable. Only peptides matching the criteria and present in the MS/MS library were considered.

At the transition level, the criteria were as follows: precursor charge: 2 and 3; fragment charge: 1; ion types: y and b; product ions ranging from (*m/z* > precursor)-1 to last ion; fragments within a 5Th window around the precursor* m/z* excluded. Both the precursor *m*/*z* and ion *m*/*z* values were restricted to span *m*/*z* range from 250 to 1400. Five most intense product ions found in the library MS/MS spectrum that comply with the above criteria were monitored. The collision energy was calculated using the slope and intercept values experimentally determined for dimethylated peptides: 0.053; −12.202 for doubly charged precursors and 0.037; −9.784 for triply charged precursors. Where appropriate both doubly and triply charged precursors for each peptide were tested even if an MS/MS spectrum for just one precursor was available in the library.

Peptides were then screened for detectability in development amniotic fluid samples using the Agilent 1260 LC system coupled to an Agilent 6490 Triple Quadrupole mass spectrometer, controlled by MassHunter acquisition software. For screening purposes, 8 *μ*L (approximately 8 *μ*g of protein) of amniotic fluid samples was injected onto a reversed-phase column (Poroshell 120 SB-C18, 2.1 × 100 mm, 2.7 *μ*m core-shell particles; Agilent), which was maintained at 40°C. The peptides were separated using a linear gradient ranging from 5% to 40% ACN with 0.1% FA over 50 min at a 300 *μ*L/min flow rate and electrosprayed into the mass spectrometer using a JetStream ion source. The acquisition method involved the following parameters: drying gas flow rate of 15 L/min at 200°C, nebulizing gas flow at 30 psi, sheath gas flow of 11 L/min at 250°C, 4000 V capillary voltage, 300 V nozzle voltage, 380 V fragmentor voltage, 4 V cell accelerator voltage, and MS operating pressure of 5 × 10^−5^ Torr. All transitions were monitored in positive ion mode with a dwell time of 10 ms and with Q1 and Q3 set to unit resolution.

Peptides detected as single chromatographic peak formed by overlapping transition signals, showing high similarity degree of transition signals with peak intensities in the MS/MS spectra, were considered for final SRM method. These peptides were subsequently used for retention time prediction, which was done for peptides with Arg and Lys separately because two methyl groups on Lys residues significantly influence retention on reversed-phase. Additional peptides detected in a predicted retention time window, with overlapping transition signals and with high similarity degree of transition and MS/MS signals, were considered as well. For each protein, the most intense precursor of three different peptides was included in the final LC-SRM method. Peptides without Met and Cys, peptides without Gln at N-terminus, peptides without Asn in a close proximity to a Gly, peptides with Lys at N-terminus, and doubly charged precursors of Arg peptides were strongly preferred. Up to five best-performing transitions were selected* per* precursor. Eventually, corresponding transitions for formaldehyde-d_2_ labeled forms were added into the final LC-SRM method.

### 2.11. Final LC-SRM Analysis and Data Analysis

Final amniotic fluid analyses were performed in two technical replicates, using 8 *μ*L of processed amniotic fluid* per* replicate (~4 *μ*g of peptides). Following each amniotic fluid sample, a standard peptide mixture and trifluoroethanol in ACN (1 : 1) were each injected once to assess system stability and to wash the injection needle and loop. The final LC-SRM analysis was performed in the scheduled manner. Individual peptide transitions were monitored within ±30 s window around the peptide retention time. The total cycle time was set to 500 ms. The maximum number of concurrent transitions was 57 and minimum and maximum dwell times were 7.34 ms and 82.05 ms, respectively. The remaining LC and MS parameters and settings were identical to those used for the initial screening for surrogate peptides.

Recorded LC-SRM chromatograms were evaluated in Skyline software. The peak integration was done automatically using Savitzky-Golay smoothing. All peaks were manually inspected to confirm proper automatic selection and integration. Maximum of two most intense transitions* per* precursor free from matrix interferences was used. Statistical evaluation was carried out in GraphPad Prism 5.03 (GraphPad Software, La Jolla, CA). Only peptides showing RSD of the total absolute intensity of heavy forms across all injections below 20% were kept. For these peptides, total precursor ratio to GIS was calculated as a weighted mean using GIS transition peak areas as the weights. Final total precursor ratio to GIS was a mean of two replicates injected. If more than one peptide per protein was monitored, total protein ratio to GIS was calculated as mean of individual final total precursor ratios to GIS. A concordance of final individual total precursor ratios to GIS was assessed in these cases. Samples with low concordance between individual precursor ratios to GIS (i.e., RSD was higher than 20%) were excluded from statistical analysis. If more than 30% of samples showed low concordance of individual precursor ratios to GIS, ratios were evaluated individually for each peptide for confirming trend observed in exploratory phase but were not allowed to be evaluated using statistical tests. The Mann-Whitney *U* test was employed for statistical evaluation. For proteins with significantly changed abundance due to MIAC and HCA, receiver-operator characteristics (ROC) were determined.

## 3. Results

### 3.1. Demographic and Clinical Characteristics of the Exploratory Cohort

The minimum number of samples for the exploratory phase (*n* = 19 in both groups) was derived based on the results of our previous studies [20]. However, because of rather limited number of samples available for the study, we included all samples that fulfilled the specified criteria: 31 MIAC and HCA-positive and 26 negative samples from women with sPTB. Table S1 presents the demographic and clinical characteristics of the women and newborns relative to the presence and absence of MIAC and HCA (see Table S1 in Supplementary Material available online at http://dx.doi.org/10.1155/2015/343501).

### 3.2. Exploratory iTRAQ Proteomic Analysis

The four-dimensional nature of the analysis led to recording of 26.230 MS/MS spectra, identifying 10.864 distinct peptides at a maximum 1% FDR. Based on these peptides, 690 amniotic fluid proteins were identified at a maximum 1% FDR. Of these, 22 noncontaminant proteins significantly differed (*P* ≤ 0.05) in both replicates in the same trend (Supplementary Materials (Table S2)). Only a single noncontaminant protein was revealed to be changed at a significance level of *P* ≤ 0.05 in decoy comparisons (iTRAQ 116/114 and iTRAQ 115/117) which allowed us to estimate the multiple comparisons FDR to be less than 5%.

### 3.3. Generating a List of Protein Candidates for Initial Verification

A candidate list for subsequent verification was compiled from proteins showing significantly altered abundance, potentially due to the presence of MIAC and HCA. The candidates were selected using the following criteria: only proteins with an identification FDR lower than 1% that showed a statistically significant change in abundance (*P* ≤ 0.05) and no discrepancy in trend were considered; proteins had to be identified using at least 5 peptides; potential contaminations and proteins targeted via immunodepletion were not considered. The 16 protein candidates were sorted according to the extremity of the change observed, calculated as the absolute log_2_ of the average iTRAQ ratio (Table 1).

### 3.4. Verification Using LC-SRM

To facilitate development of a LC-SRM assay for verification of the exploratory iTRAQ findings, we first compiled an MS/MS spectra library of dimethylated peptides from which appropriate surrogate peptides were selected. For all but filaggrin at least one peptide matched to an MS/MS spectrum and totally 106 nonrepeated peptides were assigned to an MS/MS spectrum. All peptides found for polyubiquitin-C shared sequence with polyubiquitin-B, fusion proteins ubiquitin-60S ribosomal protein L40, and ubiquitin-40S ribosomal protein S27a. Because these proteins were reported by ProteinPilot in the same protein group with almost identical score and are of the same functionality they were kept in the Skyline document. Peptides of neutrophil defensin 3 (shared with neutrophil defensin 1) and actin, cytoplasmic 1 (shared with actin, cytoplasmic 2), were treated in the same way. Two of three peptides of lipocalin-1 had shared sequences with putative lipocalin-1-like protein 1. Existence of this protein is however very uncertain (information available in the neXtProt database) and peptides were thus kept for subsequent refinement. As a result, 95 peptides were screened for detectability in immunodepleted but additionally not fractionated development samples using 524 transitions of 112 precursors. Based on the obtained intensities and preferences towards most reliable peptides we set up the final method covering 15 proteins using 38 peptides, 76 precursors, and a total of 354 transitions.

Seven peptides did not provide any transition free of interference and two peptides showed low signal stability of the heavy form. Transitions used for quantification are listed in Table S3 in Supplementary Materials. Four proteins were assayed using single peptide, 8 proteins using two peptides and three proteins using three peptides (Table 2). Peptides of collagen alpha-1(I) chain showed the worst concordance between final total precursor ratios to GIS as RSD did not achieve values below 20% in any sample. Only three samples showed satisfactory concordance of final total precursor ratios to GIS for neutrophil defensin 3. For all remaining proteins quantified using more than single peptide, at least 70% of samples showed satisfactory concordance.

**Figure 3:**
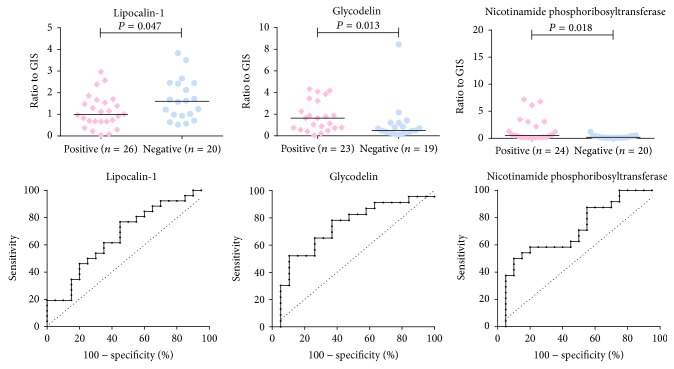
Amniotic fluid concentration of lipocalin-1, glycodelin, and nicotinamide phosphoribosyltransferase relative to GIS and respective ROC curves. In concordance with the iTRAQ exploratory findings, women without MIAC and HCA had higher amniotic fluid lipocalin-1 levels than women in whom both conditions were confirmed whereas levels of glycodelin and nicotinamide phosphoribosyltransferase were higher in women with MIAC and HCA. Some measurements did not pass the rigorous evaluation criteria for reporting only the most confident data, leading to slightly different numbers of observations subjected to statistical analysis.

From 15 proteins analyzed in individual samples using LC-SRM coupled with dimethyl labeling, data for all but two proteins were admitted to statistical analysis (Table 2). In line with the exploratory data, lipocalin-1 (P31025) showed higher level in samples from control cases (positive for MIAC and HCA: median 0.99, IQR 0.67–1.57; negative for MIAC and HCA: median 1.61, IQR 0.95–2.44; *P* = 0.047). Area under ROC curve was 67% (*P* = 0.046). Glycodelin (P09466) and nicotinamide phosphoribosyltransferase (P43490) were confirmed to be significantly more abundant in samples from women positive for MIAC and HCA (positive for MIAC and HCA: median 1.64, IQR 0.73–3.23; negative for MIAC and HCA: median 0.49, IQR 0.29–1.21; *P* = 0.013 and positive for MIAC and HCA: median 0.55, IQR 0.16–2.84; negative for MIAC and HCA: median 0.19, IQR 0.10–0.43; *P* = 0.018). Area under ROC curve for glycodelin was 73% (*P* = 0.013) and 71% (*P* = 0.017) for nicotinamide phosphoribosyltransferase (Figure 3). Logistic regression followed by ROC of combination of all three proteins yielded AUC 76% (*P* = 0.0075). Statistical analyses of data related to remaining proteins did not show significant trend in the abundance change (Table 2).

## 4. Discussion

Development of diagnostic tests to reduce inflammatory complications linked to sPTB is highly topical for decreasing overall perinatal morbidity. Previous biomarker discovery studies, addressing proteome alterations in amniotic fluid from sPTB patients with and without intra-amniotic infection and inflammation, were performed using samples obtained transabdominally at admission, that is, shortly after diagnosing preterm labor [21–23]. Potential markers (e.g., neutrophil defensins, calgranulins) and the differences between the amniotic fluid proteomes discovered using SELDI-TOF mass spectrometry and the PF2D liquid chromatography system reflect a rather early stage of inflammation, where neutrophils, as the first-line defense cells, play a fundamental role. However, information about differences in the amniotic fluid proteome at the end of labor, when activation of prolabor inflammatory pathways may diminish the response to infection, is lacking. Sampling amniotic fluid from the forebag when the cervix is fully dilated prior to artificial rupture of membranes is an effortless and noninvasive procedure compared to transabdominal amniocentesis. Thus, dysregulated amniotic fluid proteins from this stage of labor might serve as readily available potential markers for peripartum or early postpartum detection of fetuses jeopardized by the presence of intra-amniotic infection and inflammation, potentially providing insights into mechanistic aspects of sPTB complicated by MIAC and HCA.

**Figure 4:**
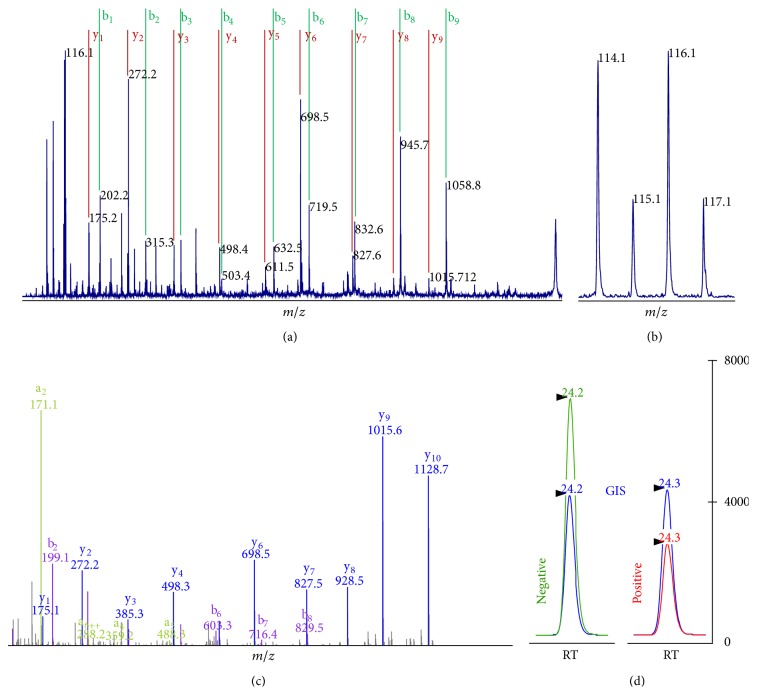
Global and targeted quantification techniques used for the exploratory and verification phase as demonstrated on lipocalin-1 peptide GLSTESILIPR. (a) MALDI-TOF/TOF MS/MS spectra of iTRAQ-labeled peptides were used for protein identification and (b) for global quantification based on the iTRAQ reporter ions 114–117. (c) For LC-SRM verification, a library of HCD MS/MS spectra of dimethylated peptides was compiled first. SRM transitions for each peptide were then selected based on the spectral information in the library (e.g., peptide GLSTESILIPR was quantified by fragments y_9_ and y_10_). (d) For accurate quantification, intensities of LC-SRM peaks obtained on QqQ instrument were normalized against a global internal standard (GIS) prepared by reaction of the same peptides with formaldehyde-d_2_.

To describe and confirm the changes in the amniotic fluid proteome related to the presence or absence of both MIAC and HCA at the end of the first stage of sPTB we employed global iTRAQ and targeted LC-SRM quantification techniques in a two-stage MS-based workflow (Figure 4 and Supplementary Materials (Figure S1 and Figure S2)).

First, we performed an exploratory analysis in representative amniotic fluid samples and employed CysTRAQ quantitation technology in combination with a multidimensional fractionation and separation, as described previously [13, 20]. The use of several levels of separation and the generation of a relatively high number of peptide fractions led to the identification of a significant number of proteins and of low-abundance proteins, as well as to more precise sample quantitation, as the coisolation issue related to iTRAQ tagged peptides is less pronounced in fractions with reduced complexity [24]. In addition, the pooling approach enabled us to perform the first phase of the study using less clinical material, which was important matter as transvaginal amniocentesis at the end of the first stage of spontaneous preterm labor is nowadays not a common procedure. Such samples are therefore rare, and a limited amount of material was available for the whole study.

The obtained set of 690 amniotic fluid proteins identified at a 1% FDR (1019 proteins at a 5% FDR) represents one of the most detailed descriptions of the amniotic fluid proteome. Proteins assessed previously in amniotic fluid using ELISA in the tens of ng/mL (myeloperoxidase) or few ng/mL ranges (cathelicidin and M-CSF) were among the proteins identified at a 1% FDR [20, 25, 26]. The number of proteins with changed abundance was rather low and did not resemble our previous findings based on the analysis on amniotic fluid in women with preterm prelabor rupture of membranes with and without both MIAC and HCA, albeit performed according to the same proteomic workflow [20].

In the second phase, we attempted to verify exploratory findings using LC-SRM in each individual amniotic fluid sample to convert the proteomic significance to a biostatistical significance. Rather than developing of the LC-SRM assay using synthetic stable isotope labeled peptides, we employed reductive dimethyl labeling of peptides using either formaldehyde or formaldehyde-d_2_. This straightforward and cost-effective technique for introduction of stable isotopes into peptides has been used for protein quantification in exploratory proteomic studies [27], but recently it has been rigidly validated for coupling with LC-SRM for targeted analyses too [16]. Using this approach we quickly generated a global internal standard for accurate relative quantification of theoretically any peptide in the sample. To accelerate the development and refinement of SRM transitions used for quantification we compiled an MS/MS library and dealt only with peptides confirmed to be present in the samples. On the other hand, GIS prepared by dimethyl labeling did not allow us to validate the assay according to guidelines for bioanalytical methods. Instead, all recorded LC-SRM data were subjected to rigorous evaluation to report only the most confident data.

Although the nanoconfiguration is still preferred in mass spectrometry-based proteomics due to higher sensitivity, we performed LC-SRM using a narrow-bore column and a standard ESI probe. The advantages of a higher flow rate and standard ESI in terms of separation reproducibility and ionization stability represent a very compelling alternative, especially when focusing on a high-throughput protein quantitation platform [28]. Using this configuration, we achieved excellent reproducibility of retention time of peptides in the test injections, which allowed us to monitor particular transitions within ±30 s window in the final method. Such tolerance coped with the isotopic effect of more hydrophilic deuterium atoms as for 90% of peptides both light and heavy forms eluted within 2.4 s and the highest difference between light and heavy form was 7.8 s for glycodelin peptide HLWYLLDLK (Supplementary Materials (Figure S1, Panel D)). RSD of retention times of all peptides was 0.2%. In addition to retention time stability, higher flow rate together with using ESI probe is believed to provide more stable signal across injections, and thus this allowed us to use total absolute intensity of peptide heavy forms as criterion for assessment of limit of quantification.

Among the dysregulated proteins detected in the exploratory iTRAQ analysis, neutrophil defensin 3 (P59666), which can be cleaved to produce neutrophil defensin 2, was found to be altered following the same trend as reported by Buhimschi et al. [22]. LC-SRM analysis in individual samples confirmed this trend, although, due to absent concordance between final total precursor ratios to GIS, data were not admitted to statistical test. Neutrophil defensin 3 is an antimicrobial peptide produced by neutrophils showing activity against Gram-negative and Gram-positive bacteria [29]. The increased level of neutrophil defensin 3 observed at the end of the first stage of spontaneous preterm labor reflects the inflammatory response to invading microbes and therefore presents a certain potential for peripartum diagnosis of inflammatory complications, even from transvaginally sampled amniotic fluid.

Lipocalin-1 (P31025) was found by Bujold et al. to be overproduced in amniotic fluid from women exhibiting preterm labor without intra-amniotic infection or inflammation who subsequently delivered at term [21]. Likewise, we found lipocalin-1 to be decreased in women with both MIAC and HCA in both iTRAQ discovery phase and LC-SRM phase, respectively (*P* = 0.047 and AUC = 0.67, *P* = 0.046). Lipocalin-1, also known as tear lipocalin or von Ebner gland protein, belongs to the lipocalin family, which share the ability to enclose lipophilic substances. The role of lipocalin-1 in tear fluid is well established [30, 31], but little is known about its dysregulation upon MIAC and HCA in amniotic fluid, which is likely related to its inhibitory activity against cysteine proteases and postulated involvement in the control of inflammatory processes [32].

LC-SRM on dimethylated peptides of glycodelin (P09466) confirmed its significantly increased level in amniotic fluid of women with MIAC and HCA (*P* = 0.013 and AUC = 0.73, *P* = 0.013). Glycodelin, also known as placental protein 14 or progesterone-associated endometrial protein, appears in four proteoforms with identical amino acids sequence but distinct attached N-glycans that govern its particular function. Glycodelin-A is the most abundant form in endometrial and amniotic fluid and is produced by endometrial and decidual epithelial cells under progesterone regulation. Its role encompasses maintenance of uterine environment suitable for physiologic pregnancy, particularly by suppressing the maternal immune system against the embryonic semiallograft [33–35]. Its role in the presence of MIAC and/or HCA has not been scrutinized yet. Oggé et al. reported glycodelin levels to be significantly decreased in the amniotic fluid of women with sPTB due to chronic chorioamnionitis when examining cases with gestational age of 32 weeks or less [36]. This is contrary to our data. However, our study described changes due to the intra-amniotic infection leading rather to an acute chorioamnionitis and furthermore we restricted the gestational age only as specified by the definition of preterm birth. When all samples included in the study by Oggé et al. were subjected to the analysis no statistical difference was reached due to neither chronic chorioamnionitis nor acute chorioamnionitis [36]. Our observation that glycodelin level is increased due to MIAC and HCA in amniotic fluid from the end of the first stage of sPTB followed by delivery is novel.

Nicotinamide phosphoribosyltransferase (P43490) is the third protein with increased abundance in amniotic fluid of women with MIAC and HCA that was confirmed independently in the verification phase of the study (*P* = 0.018 and AUC = 0.71, *P* = 0.01). Apart from its intracellular catalytic function in biosynthesis of nicotinamide adenine dinucleotide, promotion effects on maturation of B-lymphocytes and adipokine properties were ascribed to this protein [37, 38]. As a result, it is usually termed as pre-B-cell colony-enhancing factor 1 or visfatin. Among other tissues, nicotinamide phosphoribosyltransferase is produced in myometrium, placenta, and fetal membranes and its concentration in amniotic fluid increases with advancing gestational age [39]. Production of the protein responds to inflammatory mediators and is controlled by classic proinflammatory transcription factors NF-*κ*B and AP-1 [40]. Thus, it is not surprising that its abundance was shown to be elevated due to MIAC [39] and HCA [41]. Our study for the first time extends the capability of nicotinamide phosphoribosyltransferase to point out ongoing MIAC and HCA in amniotic fluid retrieved at the end of the first stage of sPTB too.

As outlined above, the design of the exploratory phase based on representative pooled samples can be considered a limitation of our study. However, even using the latest proteomic MS-based technologies, it would be very difficult to obtain such a detailed description of the amniotic fluid proteome across 57 samples through analysis of each sample individually. Because we are aware of constraints associated with the pooled strategy (e.g., a few outliers may significantly alter the average concentration in a pooled sample), in the second essential phase of the study, we attempted to assign biostatistical confidence to the top dysregulated proteins by means of LC-SRM. In this stage of the project, seven samples were unfortunately no longer available, which might have had an effect on the obtained statistical significances.

In the present study, we attributed certain potential for uncovering MIAC and HCA to several proteins from amniotic fluid that was retrieved at the end of the first stage of sPTB. Statistically different levels were verified for lipocalin-1, glycodelin, and nicotinamide phosphoribosyltransferase using LC-SRM on dimethylated peptides. These proteins are thus interesting targets for an independent validation in larger cohorts as potential peripartum markers for inflammatory complications in spontaneous preterm birth.

## 5. Conclusions

We revealed and verified significant change in levels of lipocalin-1, glycodelin, and nicotinamide phosphoribosyltransferase due to the presence of microbial invasion of the amniotic cavity and histological chorioamnionitis. Assessment of these proteins in amniotic fluid from women with spontaneous preterm labor sampled prior to delivery might thus help identify women and premature newborns who would benefit from specific perinatal and postnatal interventions.

## Supplementary Material

Supplementary Figure S1 and Figure S2: Global and targeted quantification techniques used for the exploratory and verification phase as demonstrated on peptides derived from glycodelin (Figure S1) and nicotinamide phosphoribosyltransferase (Figure S2).
Supplementary Table S1: Maternal and newborn characteristics stratified based on the presence and absence of both MIAC and HCA.
Supplementary Table S2: Proteins identified in the exploratory proteomic phase of the study.
Supplementary Table S3: Transitions used for quantification of prioritized candidates using LC-SRM assay.

## Figures and Tables

**Table 1:** List of protein candidates for verification.

*N* ^a^	Unused^b^	Access. #	Name	Peptides (95%)^c^	iTRAQ 115 : 114^d^	iTRAQ *P* 115 : 114^e^	iTRAQ 117 : 116^d^	iTRAQ *P* 117 : 116^e^	AVG log_2_⁡ change^f^
318	11.71	P0CG48	Polyubiquitin-C	7	0.26	3.82*E* − 04	0.27	8.06*E* − 04	1.93
374	10.00	P09228	Cystatin-SA	5	0.40	5.18*E* − 03	0.41	5.33*E* − 03	1.32
154	27.52	P31025	Lipocalin-1	22	0.41	7.30*E* − 03	0.48	1.49*E* − 02	1.17
110	35.41	P03973	Antileukoproteinase	28	2.12	2.66*E* − 04	2.01	3.96*E* − 04	1.04
438	7.70	P59666	Neutrophil defensin 3	10	1.83	3.59*E* − 06	1.84	3.67*E* − 06	0.88
225	17.96	P09466	Glycodelin	21	1.69	8.51*E* − 03	1.72	5.25*E* − 03	0.77
73	44.36	P00915	Carbonic anhydrase 1	51	0.58	4.96*E* − 02	0.65	4.82*E* − 02	0.70
167	25.81	P07358	Complement component C8 beta chain	16	0.64	2.84*E* − 03	0.61	5.30*E* − 03	0.68
8	175.57	P02452	Collagen alpha-1(I) chain	121	0.68	2.22*E* − 02	0.68	4.27*E* − 02	0.56
115	34.38	P60174	Triosephosphate isomerase	27	1.47	6.30*E* − 04	1.47	4.96*E* − 02	0.56
37	73.59	P60709	Actin, cytoplasmic 1	91	1.50	1.49*E* − 07	1.37	2.87*E* − 05	0.52
228	17.59	P43490	Nicotinamide phosphoribosyltransferase	10	1.41	1.63*E* − 02	1.45	2.32*E* − 02	0.51
16	113.54	Q9HC84	Mucin-5B	77	1.37	7.28*E* − 03	1.47	1.51*E* − 03	0.50
385	9.51	Q9BRK3	Matrix-remodeling-associated protein 8	5	0.69	2.61*E* − 02	0.73	2.71*E* − 03	0.49
238	16.30	P20930	Filaggrin	10	0.70	2.69*E* − 02	0.72	2.31*E* − 02	0.49
237	16.31	P14384	Carboxypeptidase M	14	0.78	7.30*E* − 03	0.78	4.74*E* − 03	0.35

^a^Protein rank relative to all other proteins identified in the exploratory analysis.

^b^ProteinPilot protein score.

^c^Number of iTRAQ peptides used for quantification of the protein.

^d^Average protein iTRAQ ratios (MIAC and HCA positive versus MIAC and HCA negative).

^e^Significance level for protein iTRAQ ratios.

^f^Extremity of the iTRAQ change calculated as the absolute log_2_ of the average iTRAQ ratio.

**Table 2:** LC-SRM assay results.

Acc. #	Protein name (peptide)^a^	Peptides^b^	Concordance^c^	*n* ^d^	*n* ^d^	Median ratio to GIS	Median ratio to GIS	*P* ^e^
				Positive	Negative	Positive	Negative	
P0CG48	Polyubiquitin-C	3	49	26	23	1.40	1.04	0.083
P09228	Cystatin-SA	1	—	27	23	0.53	1.09	0.321
P31025	Lipocalin-1	2	46	26	20	0.99	1.61	0.047
P03973	Antileukoproteinase	1	—	27	23	0.49	0.28	0.087
P59666	Neutrophil defensin 3	2	3	—	—	—	—	—
	(IPACIAGER)	—	—	27	23	0.15	0.07	—
	(YGTCIYQGR)	—	—	27	23	0.45	0.15	—
P09466	Glycodelin	2	42	23	19	1.64	0.49	0.013
P00915	Carbonic anhydrase 1	2	50	27	23	0.58	0.78	0.756
P07358	Complement component C8 beta chain	2	36	19	17	1.07	0.95	0.568
P02452	Collagen alpha-1(I) chain	3	0	—	—	—	—	—
	(ICVCDNGK)	—	—	27	23	0.43	0.36	—
	(VLCDDVICDETK)	—	—	27	23	0.79	0.64	—
	(SLSQQIENIR)	—	—	27	23	0.97	1.25	—
P60174	Triosephosphate isomerase	3	49	27	22	1.22	0.78	0.169
P60709	Actin, cytoplasmic 1	2	37	19	18	0.70	0.42	0.076
P43490	Nicotinamide phosphoribosyltransferase	2	39	24	20	0.55	0.19	0.018
Q9HC84	Mucin-5B	2	35	22	15	0.94	0.91	0.805
Q9BRK3	Matrix-remodeling-associated protein 8	1	—	27	23	1.07	1.06	0.832
P14384	Carboxypeptidase M	1	—	27	23	0.94	1.29	0.613

^a^In proteins showing low concordance between corresponding peptides, data for individual peptides are presented.

^b^Number of peptides used for LC-SRM quantification of particular protein.

^c^Number of samples in which concordance between corresponding peptides was below RSD of 20%.

^d^Number of samples that fulfilled all requirements and were subjected to statistical analysis.

^e^ *P* value as calculated by Mann-Whitney test.
