# Supplementary material for: Potential Peripartum Markers of Infectious-Inflammatory Complications in Spontaneous Preterm Birth
Source: Biomed Res Int. 2015 May 18;2015:343501. doi: 10.1155/2015/343501 (PMC4450245; doi:10.1155/2015/343501)
Supplement: Supplementary file 1 — Supplementary Figure S1 and Figure S2: Global and targeted quantification techniques used for the exploratory and verification phase as demonstrated on peptides derived from glycodelin (Figure S1) and nicotinamide phosphoribosyltransferase (Figure S2). Supplementary Table S1: Maternal and newborn characteristics stratified based on the presence and absence of both MIAC and HCA. Supplementary Table S2: Proteins identified in the exploratory proteomic phase of the study. Supplementary Table S3: Transitions used for quantification of prioritized candidates using LC-SRM assay. [file 343501.f1.zip › Supplementary Material_Table S1.pdf]

	The presence of both MIAC and HCA (n=31)	The absence of both MIAC and HCA (n=26)	*p*-value
**Maternal age (years)**	27.3±6.0	26.5±6.0	0.64
**Nulliparous**	14 (45%)	18 (69%)	0.11
**Smokers**	6 (19%)	6 (23%)	0.76
**Gestational age at sampling and delivery (days)**	34+6 (24+4-36+0)	34+3 (29+0-36+2)	0.15
**Birth weight (grams)**	2111±800	2238±721	0.54
**Spontaneous delivery**	19 (61%)	15 (58%)	0.79
**Cesarean section**	12 (39%)	11 (42%)	0.79
**Apgar score < 7 in 1 minute**	9 (29%)	6 (23%)	0.76
**Apgar score < 7 in 5 minutes**	0 (0%)	2 (8%)	0.20
