# Supplementary material for: Potential Peripartum Markers of Infectious-Inflammatory Complications in Spontaneous Preterm Birth
Source: Biomed Res Int. 2015 May 18;2015:343501. doi: 10.1155/2015/343501 (PMC4450245; doi:10.1155/2015/343501)
Supplement: Supplementary file 1 — Supplementary Figure S1 and Figure S2: Global and targeted quantification techniques used for the exploratory and verification phase as demonstrated on peptides derived from glycodelin (Figure S1) and nicotinamide phosphoribosyltransferase (Figure S2). Supplementary Table S1: Maternal and newborn characteristics stratified based on the presence and absence of both MIAC and HCA. Supplementary Table S2: Proteins identified in the exploratory proteomic phase of the study. Supplementary Table S3: Transitions used for quantification of prioritized candidates using LC-SRM assay. [file Supplementary Material_Figure S1 and Figure S2.pdf › Supplementary Material_Figure S1 and Figure S2.pdf]

S1

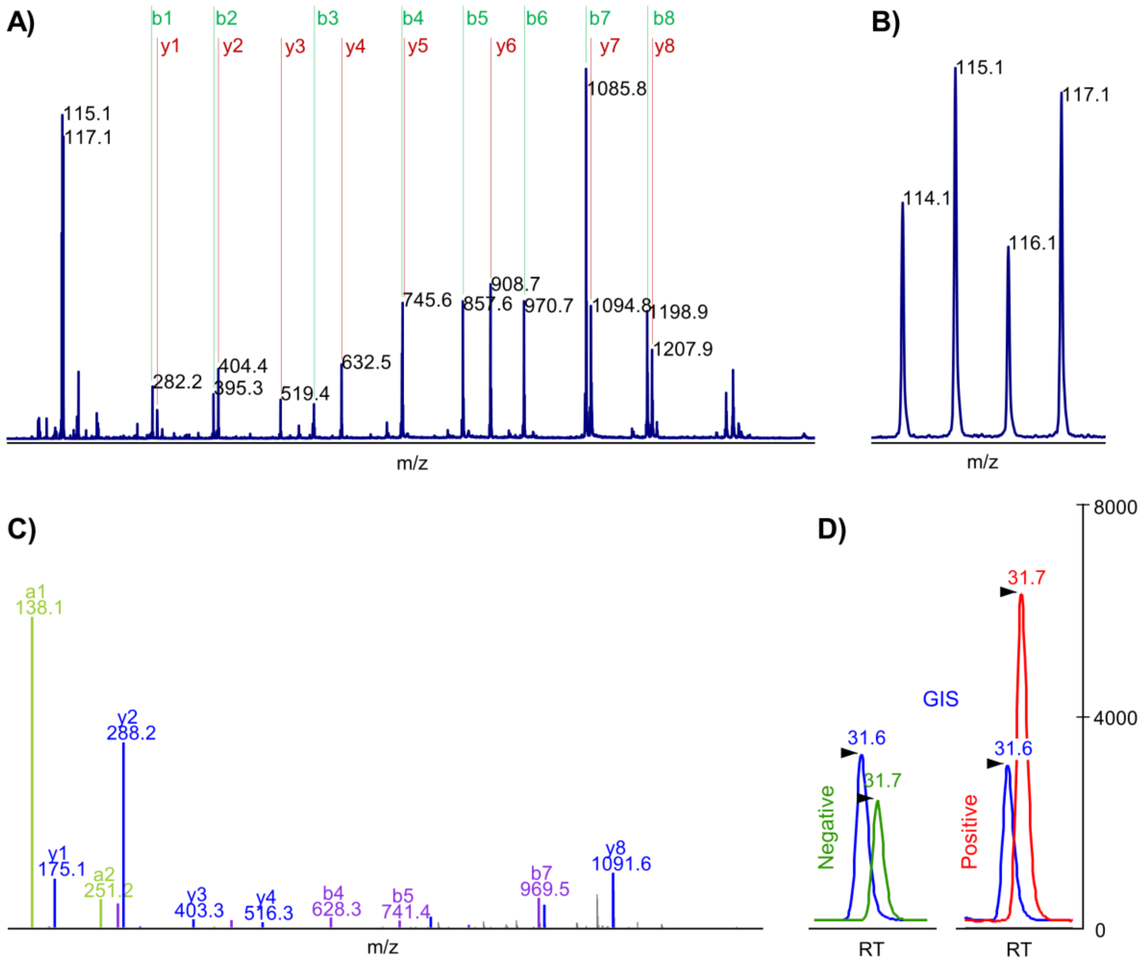
A)
b1 b2 b3 b4 b5 b6 b7 b8
y1 y2 y3 y4 y5 y6 y7 y8
115.1
117.1
1085.8
908.7
745.6
857.6
970.7
1094.8
1198.9
1207.9
404.4
395.3
282.2
519.4
632.5
m/z
B)
115.1
117.1
114.1
116.1
m/z
C)
a1
138.1
y2
288.2
y1
175.1
a2
251.2
y3
403.3
y4
516.3
b4
628.3
b5
741.4
b7
969.5
y8
1091.6
m/z
D)
8000
4000
0
GIS
31.7
31.6
31.6
31.7
Negative
Positive
RT
RT

S2

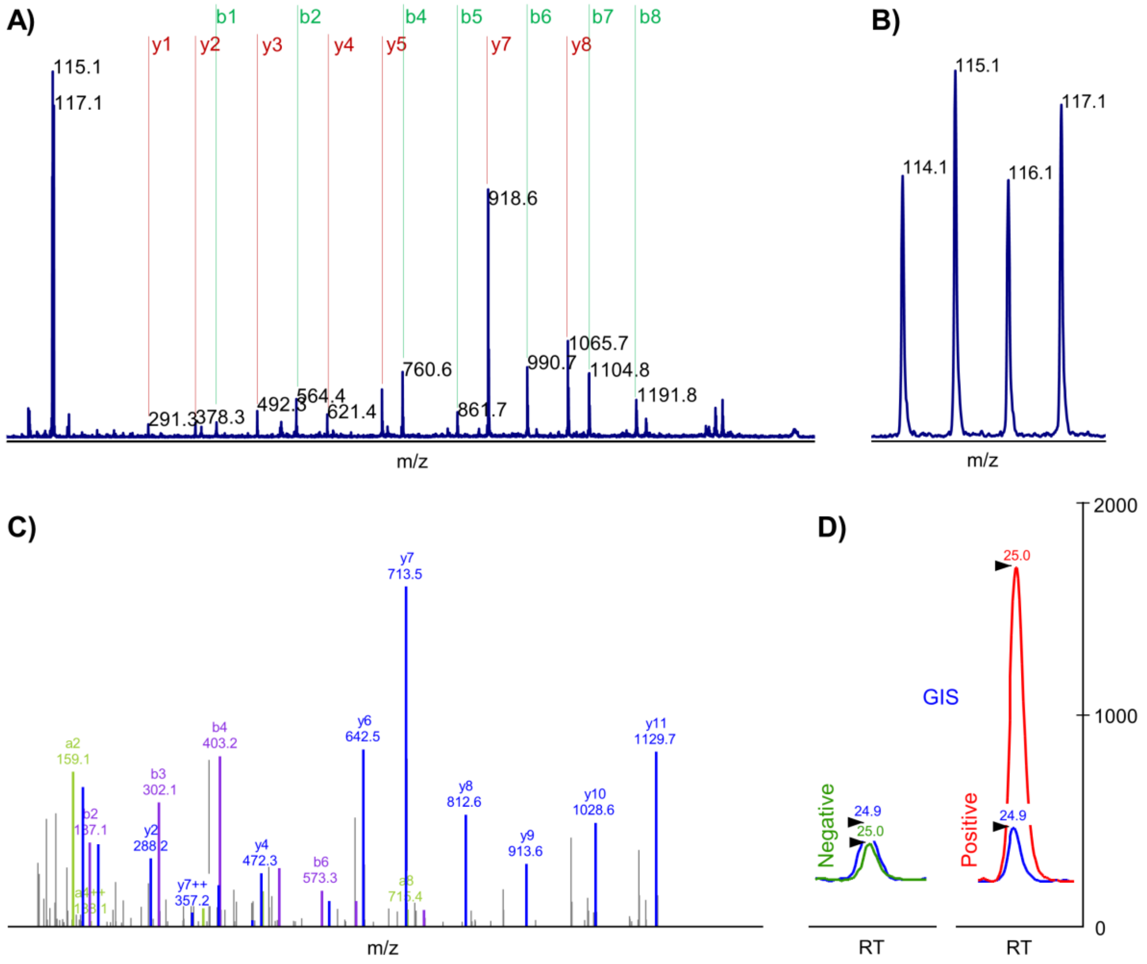
A)
b1 b2 b4 b5 b6 b7 b8
y1 y2 y3 y4 y5 y7 y8
115.1
117.1
918.6
1065.7
990.7
1104.8
760.6
1191.8
564.4
492.3
621.4
291.3
378.3
861.7
m/z
B)
115.1
117.1
114.1
116.1
m/z
C)
y7
713.5
y6
642.5
y11
1129.7
a2
159.1
b4
403.2
b3
302.1
b2
187.1
y2
288.2
y8
812.6
y10
1028.6
y4
472.3
b6
573.3
y9
913.6
y7++
357.2
m/z
D)
2000
1000
0
GIS
25.0
24.9
25.0
24.9
Negative
Positive
RT
RT
